# Contrast opacification on thoracic CT angiography: challenges and solutions 

**DOI:** 10.1007/s13244-016-0524-3

**Published:** 2016-11-17

**Authors:** Abhishek Chaturvedi, Daniel Oppenheimer, Prabhakar Rajiah, Katherine A. Kaproth-Joslin, Apeksha Chaturvedi

**Affiliations:** 1 Department of Imaging Sciences, University of Rochester Medical Center, 601 Elmwood Ave, P.O. Box no. 648, Rochester, NY 14642 USA; 2 Department of Radiology, University of Texas Southwestern, Dallas, TX USA

**Keywords:** CT angiography, Non-diagnostic CTA, Contrast dynamics, Cardiac asystole, ECMO

## Abstract

Contrast flow and enhancement patterns seen on thoracic CT angiography (CTA) can often be challenging and may often reveal more than is immediately apparent. A non-diagnostic CTA following the initial contrast injection can be secondary to many causes; these include both extrinsic factors, such as injection technique/equipment failure (iv cannula, power injector), and intrinsic, patient-related factors. Contrast pressure and flow graphs often contain useful information regarding the etiology of a non-diagnostic scan. Understanding these graphs will help the radiologist plan a repeat contrast injection to overcome the deficiencies of the first injection and thus obtain a diagnostic scan. The current review article outlines normal and abnormal intravenous contrast dynamics, discusses how to recognize etiologies of non-diagnostic scans, and ultimately addresses techniques to overcome obstacles towards obtaining normal contrast opacification of the target vessel. In addition, there are some life-threatening findings, which unless sought for, may remain hidden in plain sight.

*Key Points*

• *Using contrast enhancement and flow patterns to identify the cause of a non-diagnostic CTA*.

• * Recognize life threatening causes of altered contrast dynamics such as cardiac asystole*.

• *Non-target vessel opacification may hold key to underlying pathophysiology*.

**Electronic supplementary material:**

The online version of this article (doi:10.1007/s13244-016-0524-3) contains supplementary material, which is available to authorized users.

## Introduction

There is progressively increasing use of CT scans in the emergency department (ED) in recent years [1]. Chest pain is the second leading presenting symptom in ED patients (5.2 %) [2], thus thoracic CT angiography (CTA) has become one of the most commonly ordered CTA in ED. Indications for thoracic CTA include pulmonary embolus, acute aortic syndrome, or coronary artery disease. Contrast flow and enhancement patterns seen on CTA can often be challenging and may often reveal more than is immediately apparent. In addition to target vessel opacification, evaluation of non-target vessels may also contain important clues to the underlying disease that brought the patient to the ED. In addition, there are some life-threatening findings, which unless sought for, may remain hidden in plain sight. Also, contrast pressure and flow graphs obtained with the CTA often contain useful information regarding the etiology of a non-diagnostic scan (Table 1). Understanding these graphs will help the radiologist plan a repeat contrast injection to overcome the deficiencies of the first injection and thus obtain a diagnostic scan.

**Table 1:** Different causes of abnormal contrast flow, key imaging findings and solutions to obtain a diagnostic CTA

Etiology of altered contrast flow	Key imaging features	Solution
Access cannula and vein mismatch	1. Suboptimal target vessel opacification 2. Injection flow rate may be lower than planned	1. New cannula or access vein 2. When access veins are small: Dual energy scan with 50–60 keV monoenergetic reconstruction
Incorrectly placed region of interest	1. Assess location of region of interest from the bolus tracker/ bolus timing images	Correct placement of ROI, reinjection, and reimage
Extravasation	1. No target vessel opacification 2. Contrast presence in soft tissues of access vein extremity	1. New cannula and access vein at a different site
Thoracic venous outlet obstruction	1. Contrast pooling in the collaterals around axilla & chest wall	1. Asymptomatic: reinjection with arm down position 2. Symptomatic: new access site in contralateral extremity
Transient interruption of contrast bolus	1. Transient decreased contrast attenuation. 2. Presence of normal contrast in upstream vessels	1. Shallow breath hold 2. End expiratory imaging 3. Free breathing high pitch acquisition
Differential enhancement in pulmonary artery		1. Delayed acquisition 2. Biphasic injection
Differential enhancement in aorta		1. Multiplanar reformats to evaluate coarctation, shunts.
Mixing artifact in aorta	Contrast blood level, dependent pooling of contrast	1. Assessment of cardiac function Repeat delayed (30 sec) limited Z axis scan
Mixing artifact in left atrium	Contrast blood level in left atrium, pulmonary veins	Assessment of cardiac function
Poor opacification of left ventricle	No opacification of left ventricle on a pulmonary artery CTA	Assessment of cardiac function
Early enhancement on left compared to right heart	Intracardiac shunt	Assessment of cardiac function
Asystole	Dependent pooling of contrast in central veins, liver	Initiate cardiopulmonary resuscitation and page the code team

In this article, we will review several abnormal contrast enhancement and flow patterns that are encountered in thoracic CTA, including severe abnormalities such as right heart strain, cardiac asystole, and cardiac tamponade. In addition, we will also revisit key components of intravenous contrast delivery including, but not limited to power injector, intravenous cannula size, flow rate, and access vein size.

## Obtaining a diagnostic thoracic CT angiography (CTA)

An optimal, diagnostic thoracic CTA study is never obtained by accident. It requires active technologist and often radiologist input throughout the planning, execution, and post-processing stages.

The physical components of a CTA include the CT scanner, intravenous cannula, access vein size, and power injector; the functional component is the patient’s cardiac status. Optimal functioning and seamless integration of every individual piece of this continuum is crucial to ensure optimal contrast opacification of the target vessel, and thus, a diagnostic study, since “*a chain is only as strong as its weakest link*”. Through the following text, we will address the important pieces of this fine-tuned sequence and how malfunction of any individual component can limit attempt﻿s to obtain a diagnostic study. With this end goal in mind, understanding the anatomy and physiology of the cardiovascular system as it pertains to contrast flow dynamics can serve as a useful starting point.

## Blood/contrast flow dynamics and cardiac function

Central veins of the thorax convey blood/contrast bolus to the right atrium. Blood then enters the right ventricle across the tricuspid valve. Further, the right ventricle propels the blood/contrast medium into the pulmonary artery from where it enters the pulmonary vein and then the left atrium. Mitral valvular opening and left atrial contraction ensure onward flow of blood/contrast bolus into the left ventricle; systolic contraction of the left ventricle further propels blood/contrast bolus into the aorta and its branches.

Normal flow of intravenous contrast through a commonly used upper extremity venous access site follows a pattern; contrast flows first into the brachiocephalic vein, then into the superior vena cava, right atrium, right ventricle, pulmonary artery, pulmonary vein, left atrium, left ventricle, ultimately to opacify the ascending and then the descending aorta.

Any disruption of the above-described normal sequence of events should be treated with suspicion. For example, contrast enhancement of a distal chamber before adequate opacification of a proximal vessel/chamber is never normal. Similarly, persistent enhancement of a proximal segment when most of the contrast has washed out from the distal segments is not normal. When present, these findings should raise the suspicion of altered flow dynamics including underlying intra- or extra-cardiac shunt.

Traditionally, ECG gated CT/MR have been used to define cardiac chamber enlargement. However, recent literature has suggested threshold measurements to identify left atrial enlargement (transverse diameter: 73 mm, anteroposterior diameter: 43 mm) [3] and left ventricle enlargement (56 mm) [4] on a non-ECG gated CTA. For the right atrium, usually the normal diameters suggested by echocardiography on four-chamber view have been used: right atrium (long-axis dimensions 3.4–5.3 cm and 2.6–4.4 cm for short axis) and for right ventricle (basal dimensions 3.9–4.5 cm and longitudinal dimension 8–9.1 cm) [5].

## Normal circulation

**Fig. 1:**
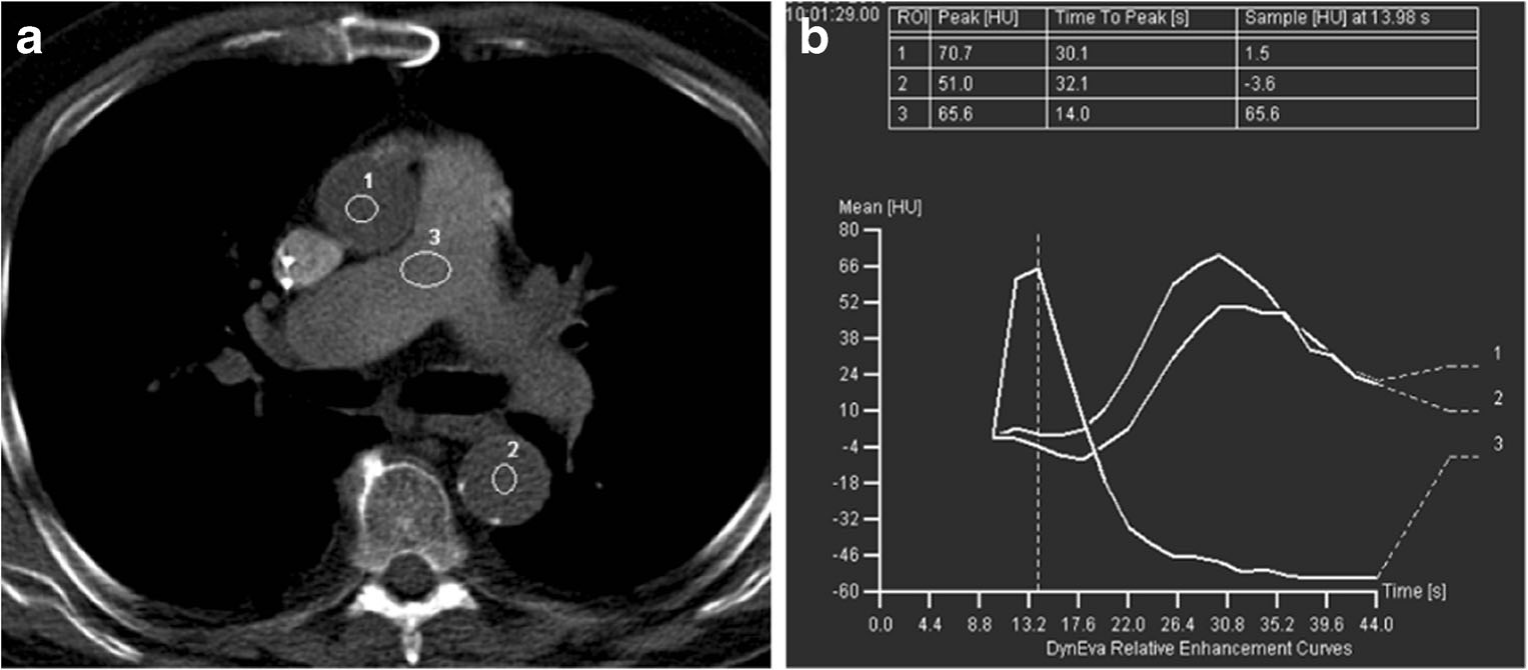
Test bolus technique for identification of contrast arrival in target vessel. Prolonged pulmonary circulation time in a patient with aortic stenosis. CT image during test bolus injection (**a**) with regions of interest over the ascending aorta (*1*), descending aorta (*2*) and pulmonary artery (*3*). Attenuation-time graph corresponding to the regions of interest (**b**) shows prolonged pulmonary circulation time of 16 s in this patient due to aortic stenosis (normal pulmonary transit time is approximately 7–9 s)

The pulmonary artery to ascending aorta transit time (PTT) is a key circulatory parameter that can affect target, as well as non-target vessel opacification on a thoracic CTA. PTT can be calculated by evaluating the time attenuation graph obtained from the test bolus (Fig. 1). In normal subjects, this is about 6.8 ± 1.7 s [6]. In patients with pulmonary hypertension or congestive heart failure (CHF), pulmonary circulation can be slow and PTT will be prolonged, which may lead to delayed contrast arrival in the non target vessel, e.g. in cases of a pulmonary artery CTA, this implies delayed aortic opacification.

## CT scan

There may be more than one type of CT scanner within the same hospital system. Understanding the type of CT scanner used is important as scan parameters such as transit time, acquisition time, applied kVp and mAs may differ between scanners. Importantly, the scan delay and total acquisition time may vary depending on the scanner (Table 2). These parameters can be altered to reduce contrast volume in patients with diminished renal function or when a repeat injection becomes necessary in event of an initial, non-diagnostic scan. When using a scanner with shorter acquisition time, non-target vessel enhancement may be less than expected and these vessels should be interpreted with caution.

**Table 2:** Key differences in the different parameters of the commonly used CT scanners

	Collimation	Pitch	Gantry rotation time (s)	Scan delay time (s)	Total scan time (s)
64 slice	64 × 0.625	0.6–1	0.75	4.2	7–9.4
128 slice	64 × 0.625	0.6–1	0.40	4.2	4–7
256 slice	128 × 0.625 × 2	0.6–1	0.33–0.4	4.2	3–6
Dual source FLASH	2 × 128 × 0.625	1–3.2	0.28	3–6.0	1.1–2

Imaging pearl: Know the transit delay and scan acquisition time of the CT scanner. Modify the injection protocol and post-threshold delay based on these factors. Post-threshold delay needs to be increased when using a faster scanner to better opacify the non target vessels. For example, for a pulmonary embolus study, we use a post-threshold delay of at least 5 s on a 64 slice scanner, but a longer delay of 8 s is used on a 256 or a dual source scanner.

## CTA techniques

Contrast arrival in the target vessel can be determined by using test bolus or bolus tracker technique [7, 8]. Evaluating the time attenuation curves generated by either of these techniques is important to understand normal and abnormal contrast arrival and pulmonary transit time.

In **test bolus** technique, a small amount of contrast is injected followed by saline chaser at the predetermined flow rate to identify contrast arrival in target vessels. It assumes that the bolus geometry of this initial injection and the final injection for the diagnostic scan would remain the same (Fig. 1). In **bolus tracking** technique, only one injection is performed at the planned flow rate. The scan is initiated as soon as the contrast enhancement threshold (predetermined threshold HU) is reached within the region of interest. The time taken from when this threshold is achieved to the actual beginning of the diagnostic scan depends on multiple factors including the scanner type and distance of this bolus tracker location with reference to the first slice of the acquisition. This is called the transit delay (Table 2) and varies from patient to patient and scanner to scanner.

**Fig. 2:**
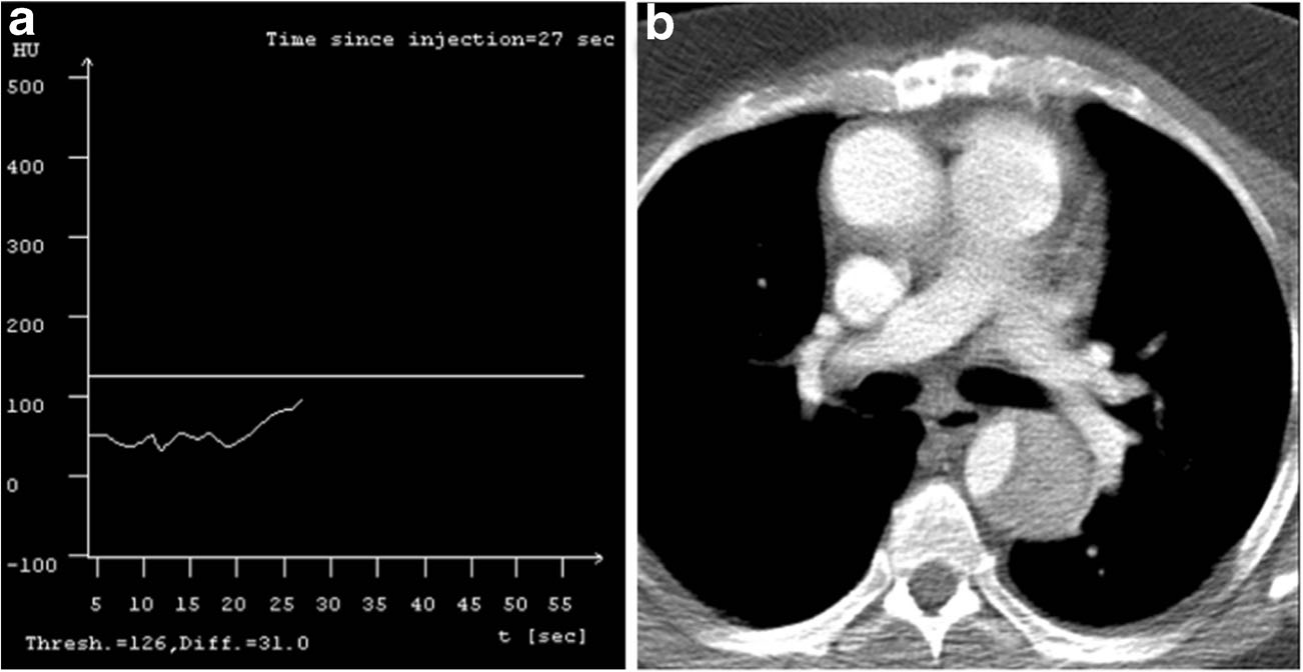
Bolus tracking technique for identification of contrast arrival. Patient with descending aortic dissection with suboptimal increase in contrast opacification within the region of interest (ROI) and failure to trigger the scan (**a**). The initial CTA ROI was placed in the false lumen (**b**). The contrast opacification of the false lumen does not reach the threshold required to trigger the scan as seen on on bolus tracker HU-time graph. Repeat injection with placement of ROI in true lumen resulted in adequate evaluation of dissection

Both these techniques, however, depend on correct identification of target vessel to place the region of interest where change in attenuation is being measured. If this location is incorrect, such as a false lumen of an aortic dissection, the attenuation may not reach the threshold and the scan may not be initiated (Fig. 2).

Imaging pearl: In patients with known heart failure, test bolus can be more useful in identifying time to peak enhancement, which can be delayed compared to contrast arrival time.

## Intravenous cannula

Size of the cannula used for delivery of contrast medium is important to achieve the desired flow rate for the duration of the injection [9]. Peak flow rate needed varies on the indication for the CTA: preferred flow rate for pulmonary artery CTA is 3–5 cc/s [10], for aortic evaluation is > 3 cc/s [11], and for coronary artery assessment is > 5 cc/s [12] (Table 3).

**Table 3:** Peak flow rate used at our institution based on manufacture recommendations and ACR guidelines

Type of line	Injection rate max	PSI rating	Hand injection	Power injectable
18 g	5 cc/sec	300	YES	YES
20 g	4 cc/sec	300	YES	YES
22 g	1 cc/sec	300	YES	YES
24 g	Not recommended	N/A	YES	NO

## Access vein

**Fig. 3:**
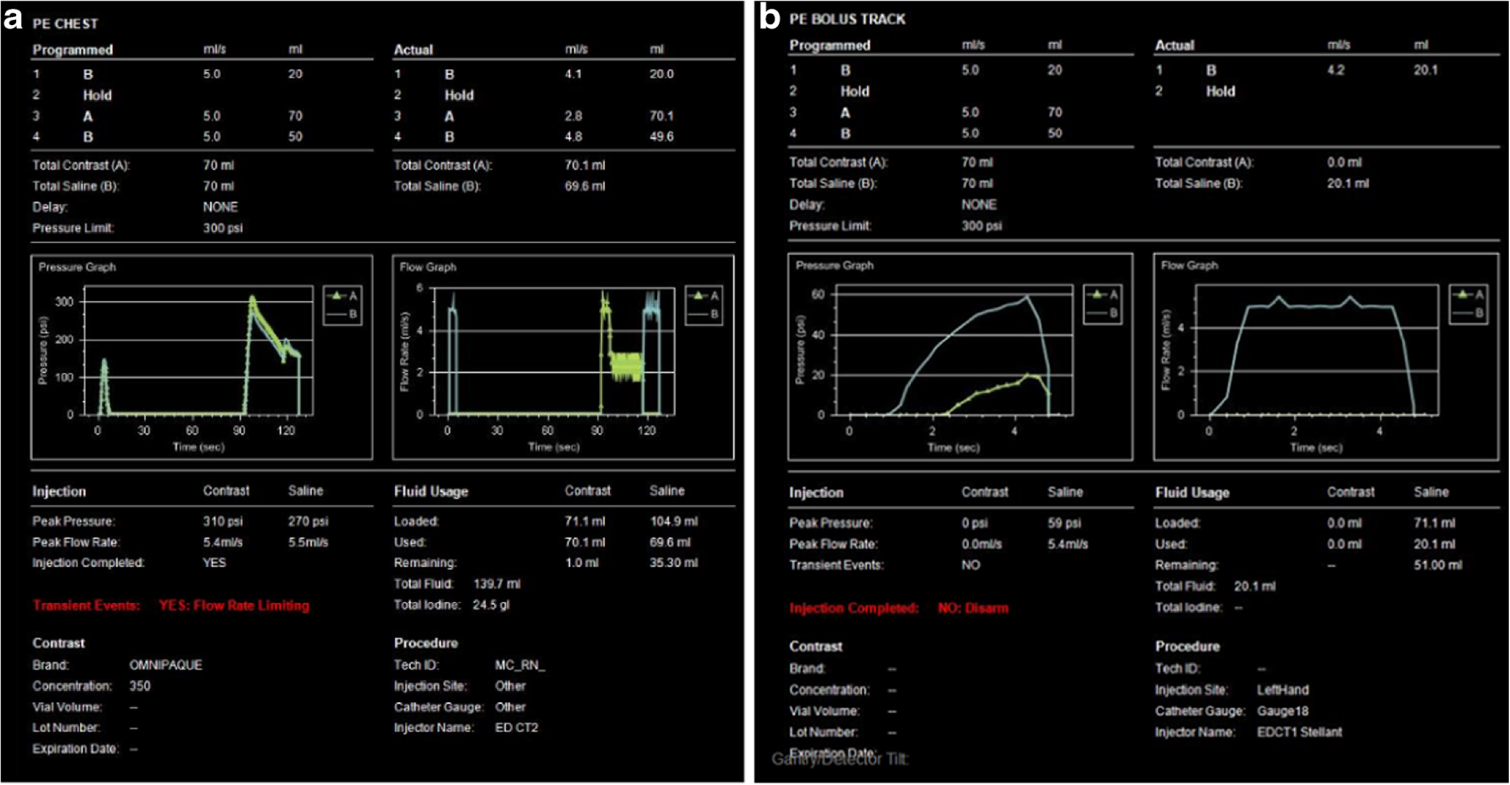
Graphs and tables from the power injector: pressure-time and flow rate-time graphs. In a patient with contrast flow rate above the recommended maximum (**a**). The planned flow rate of 5.4 mL/s using an 18 g IV exceeded the recommended maximum of 5.0 cc/s. This resulted in peak pressure reaching 300 psi with resultant disarming and decreased flow rate of 2.8 mL/s with suboptimal pulmonary artery enhancement. Power injector pressure-time and flow rate-time graphs in a different patient with IV infiltration during the injection (**b**) shows rapid drop in pressure and cessation of flow at the time of contrast extravasation

The peak flow rate that can be achieved also depends on the size of the access vein [9] (Table 4). It is important to identify this before the injection is initiated as a mismatch can lead to either power injector induced reduction in flow rate of the injection due to peak pressure being reached (Fig. 3a), leak at the level of the hub (Fig. 3b) with abrupt cessation of flow, or contrast medium extravasation.

**Table 4:** Peak flow rate for contrast medium injection based on access vein

Location	Flow rate
Hand/wrist/venipuncture	1.5 mL/s
Antecubital/forearm	All power injection
Central venous catheter*	<2.5 mL/s

## Power injector

Power injector is used to inject contrast medium and saline chaser at a constant flow rate for the entire duration of the injection. Two graphs are generated by the power injector, which plot the change in pressure over time and flow rate of the injection over time (3 A, B). Evaluation of these graphs is important in identifying the planned flow rate and any changes to that. In case of an access vein size and i.v. cannula mismatch the pressure may exceed the threshold, which may limit the flow rate. This has important implications for a diagnostic scan, especially pulmonary CTA as the injection may not occur at the peak rate planned thus leading to suboptimal opacification. Interpretation of these graphs can help identify the cause of a nondiagnostic scan in the first place and what parameters need to be changed before we plan a reinjection.

Imaging pearl: Check the access vein and access cannula before initiating contrast injection. In patients with small caliber access veins, a dual energy acquisition can be obtained. Lower (40–60 KeV) monoenergetic reconstruction can be obtained to boost contrast opacification. When a dual energy scanner is not available, we use a larger volume (1.5 mL/kg) of contrast medium containing 350 mgI/mL iodine and use 80 or 100 kVp for image acquisition.

## Technical causes of poor contrast opacification of target vessel of interest

### Incorrectly placed ROI

Inappropriate placement of ROI for bolus tracking scan is a common cause of non-diagnostic CT scan. Human error is a common source of inappropriate placement. Selection of the wrong target vessel, especially in the setting of complex vascular anatomy, and/or selection of an ROI which is too big or too small are common operator dependent errors. Patient movement between localizer slice selection/ROI placement and the start of contrast administration/imaging can also affect ROI placement (i.e. large respiratory effort, cardiac motion, and/or the patient physically shifting on the table), leading to premature, delayed or even no bolus triggering. In addition, intraluminal abnormalities, including dissection and embolus, may not be readily apparent on the precontrast localizer images and placement of the ROI overlying one of these structures may result in delayed or absent bolus triggering. For example, if the ROI is placed in the false lumen of a type B aortic dissection (Fig. 2), the contrast enhancement may or may not rise as quickly as expected (2). To avoid this, the indication of the scan should be well known to the operating technologist. A test bolus is preferable to bolus tracking in patients with post-surgical repair of complex congenital heart diseases.

Imaging pearl: In patients with known aortic dissection, test bolus can be more useful in identifying time to peak enhancement in true and false lumens. Optimal time for acquisition would be when both lumens are opacified. If false lumen dose not opacify at all on the bolus timing scan, a limited Z axis 60 s delayed image can be obtained to confirm slow flow/ thrombus or for follow-up, contrast-enhanced MRA may be obtained.

### Extravasation

**Fig. 4:**
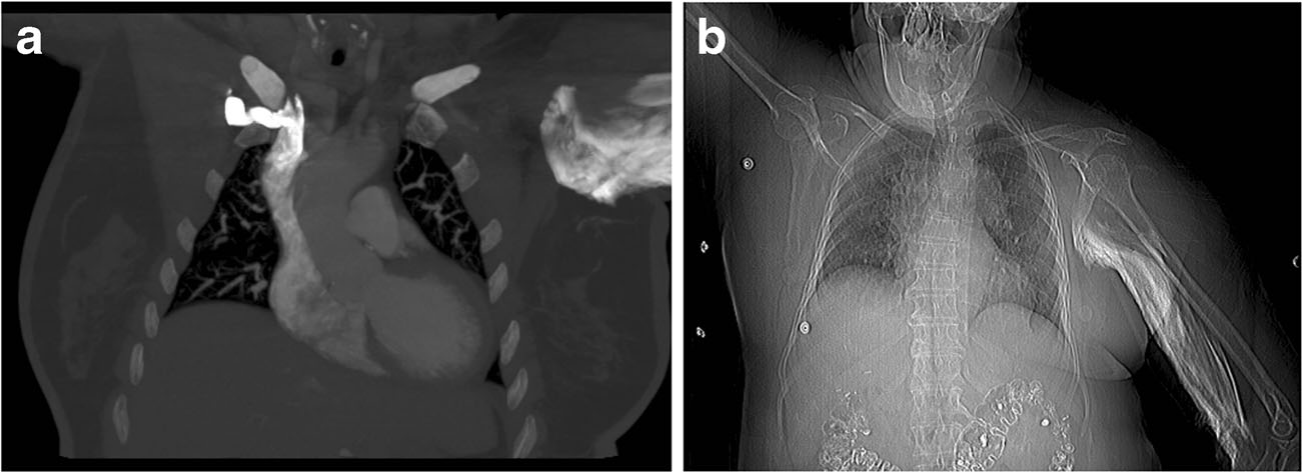
**a** Extravasation of intravenously injected contrast material. Coronal reformatted CECT images depicts extravasated contrast in the upper extremity soft tissues. **b**: CT scout image after extravasation of intravenously administered contrast in the left upper extremity allows assessment of the extent of extravasation and permits evaluation of possible compartment syndrome

Extravasation of contrast material, in which contrast medium is injected outside the intended vessel, is an infrequent, but well known complication of CTA (Fig. 4a). Contrast extravasation rates during CT imaging range between 0.1 and 0.9 %, with an average rate of 0.4 % [13, 14]. Patients at risk for contrast extravasation include infants and small children, elderly, uncooperative, and unconscious patients, as they may not be able to communicate or complain of pain reliably during injection. Patients receiving chemotherapy also have an increased risk due to fragile, damaged, and often small caliber vessels. In addition, use of distal access sites (i.e. hand or foot), use of power injection, use of a vessel with multiple puncture attempts, and use of a peripheral IV that has been in place >24 h can also increase the risk of extravasation [13–15]. Contrast extravasation should be considered if the power injector demonstrates unexpected rapid drop in pressure or exceeds the pressure limit with sudden decrease in flow rate before the full volume of contrast is administered to the patient. Radiograph or CT topogram imaging of the affected limb following an extravasation event may be useful to determine the magnitude of infiltration and verify if compartmentation is present (Fig. 4b) [15]. If some contrast has gone into the patient, the study may still be salvageable. This should be reviewed by the radiologist. If contrast is suboptimal, sometimes it can be amplified by using virtual monoenergetic images from a dual energy scanner. By using low energy virtual monoenergetic images, the energy levels of which are closer to the K edge of iodine, the contrast signal is amplified which can potentially salvage some suboptimal studies. However, if the study is not salvageable or if no contrast went into the area of interest, the study will have to be repeated. This can be done immediately if there is another venous access or later after obtaining appropriate venous access.

ACR Manual on Contrast Media discusses the treatment of contrast extravasation [9]. Raising the affected limb above the level of the heart may reduce swelling and facilitate absorption of extravasated fluid. Some favor cold compresses to decrease pain at the extravasation site and others prefer warm compresses to improve blood flow to the extravasation site and increase absorption of the contrast from the tissues into the vasculature and lymphatics. In addition, attempts to remove the extravasated contrast via aspiration have not been shown to be consistently beneficial. Regardless of the post-extravasation treatment method, patients should be evaluated by the radiologist. If pain is the main symptom, we use cold compresses, and if the extravasation has occurred in a location where there is a higher likelihood of compartment syndrome, we use hot compresses. We observe the patient in the radiology department for at least 1 h to ensure that there are no new symptoms, such as pain or numbness to suggest development of compartment syndrome. Before discharge, a radiologist discusses the findings that would suggest a developing compartment syndrome with the patient. The patient is instructed to seek medical attention if new neurologic or circulatory symptoms or skin ulceration develop [9].

### Thoracic venous outlet obstruction

Thoracic outlet syndrome (TOS) refers to the effects of dynamic compression of the nerve, artery, and/or vein as these structures cross the thoracic outlet due to changes in arm position, typically induced by elevation of the arms [16]. Dynamic CTA, with the arm in neutral position and then in elevated positions (130° of hyperabduction with external rotation), can be used to evaluate TOS [17, 18]. Contrast injection should be administered into the vein of the asymptomatic extremity to reduce beam hardening artifact [17, 18].

**Fig. 5:**
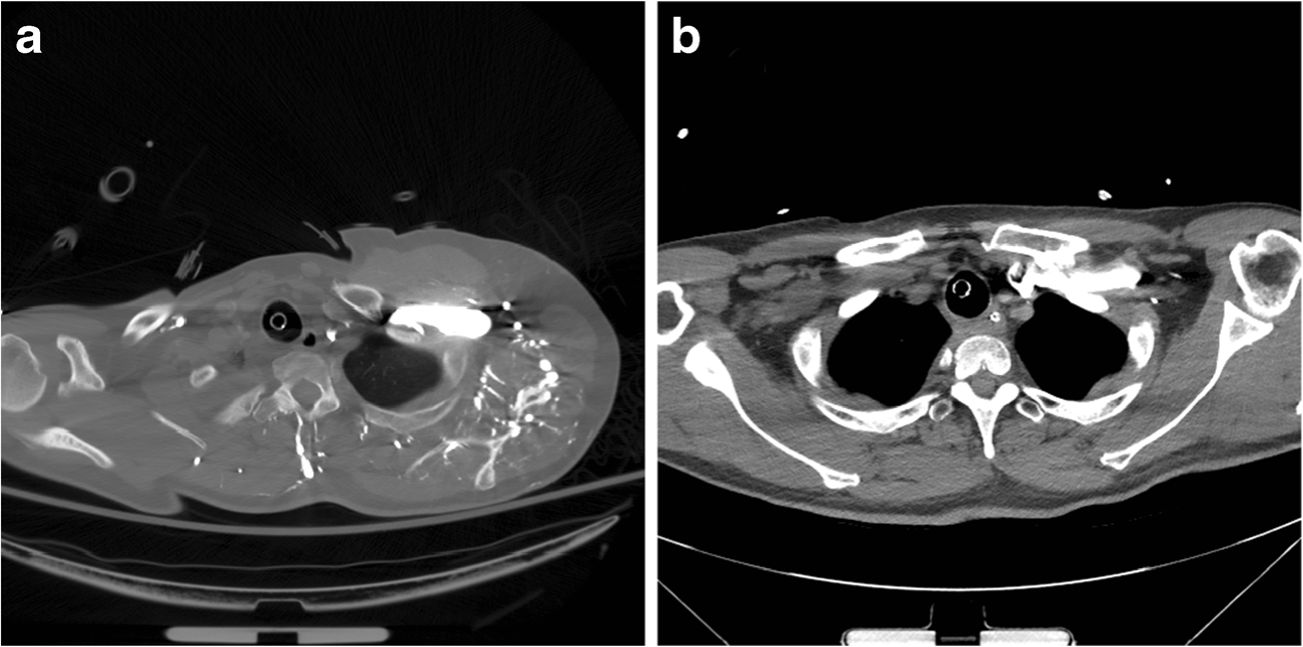
Thoracic venous outlet obstruction of the left subclavian vein with the left arm raised for CTPA, which subsequently resolves upon positioning the arm down at the side. Axial CECT following the rapid bolus of intravenous contrast in the left upper extremity with the left arm raise (**a**) shows narrowing of the left subclavian vein at the thoracic inlet and contrast filling multiple small collateral veins in the left shoulder region. The narrowing of the left subclavian vein prevented adequate opacification of the pulmonary artery. Repeat injection with the left arm down by the patient’s side (**b**) shows excellent opacification of the left subclavian vein and no filling of venous collaterals, permitting a diagnostic scan for evaluation of pulmonary embolus

**Fig. 6:**
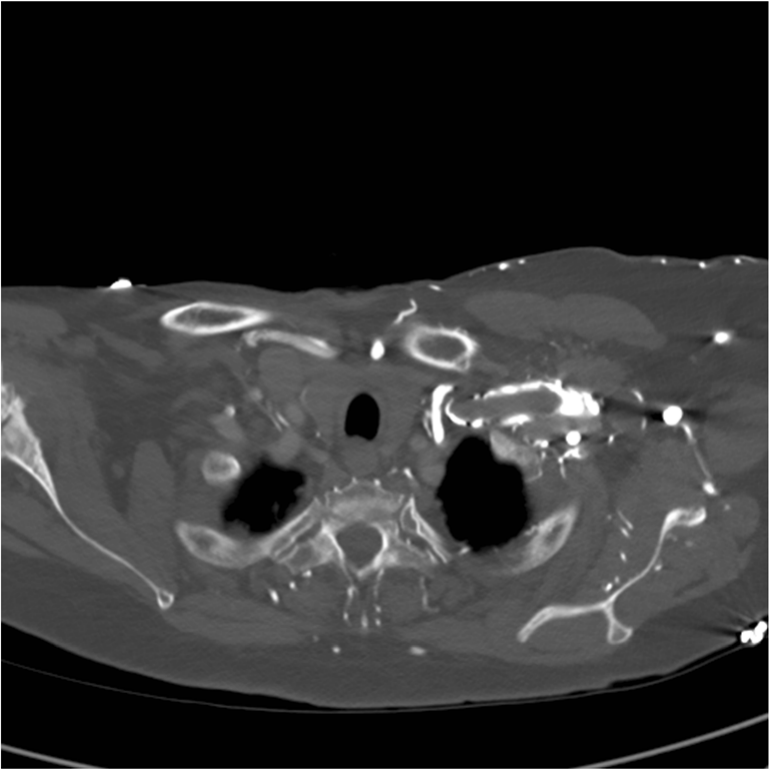
Chronic left subclavian vein thrombus with venous collateral formation. Axial CECT after injection of intravenous contrast in the left upper extremity shows a large filling defect in the left subclavian vein (*arrows*) and multiple venous collaterals in the left shoulder region. Unless the vessel is patent, additional studies should be performed with a contralateral upper extremity injection to permit adequate contrast opacification

As the majority of thoracic CTAs are performed with the patient’s arms raised, compression of the subclavian vein (asymptomatic or symptomatic) can lead to compromises in IV contrast delivery to the central vascular structures, affecting bolus timing and leading to suboptimal opacification due to reductions in flow rate (Fig. 5a). If suboptimal contrast opacification of the target vessel is present, reimaging the patient with the arm in the neutral or adducted position should relieve the dynamic narrowing of the thoracic outlet, thereby improving opacification of the vessel (Fig. 5b). Secondary signs of venous stenosis include dynamic collateral vessel filling and distal venous thrombus in chronic cases (Fig. 6). Thoracic venous outlet obstruction should be considered when extensive collateral vessel filling is seen on the side of contrast administration when the patient’s arms are raised. Alternatively, new access from the contralateral extremity vein can be obtained.

### Transient interruption of the contrast bolus

**Fig. 7:**
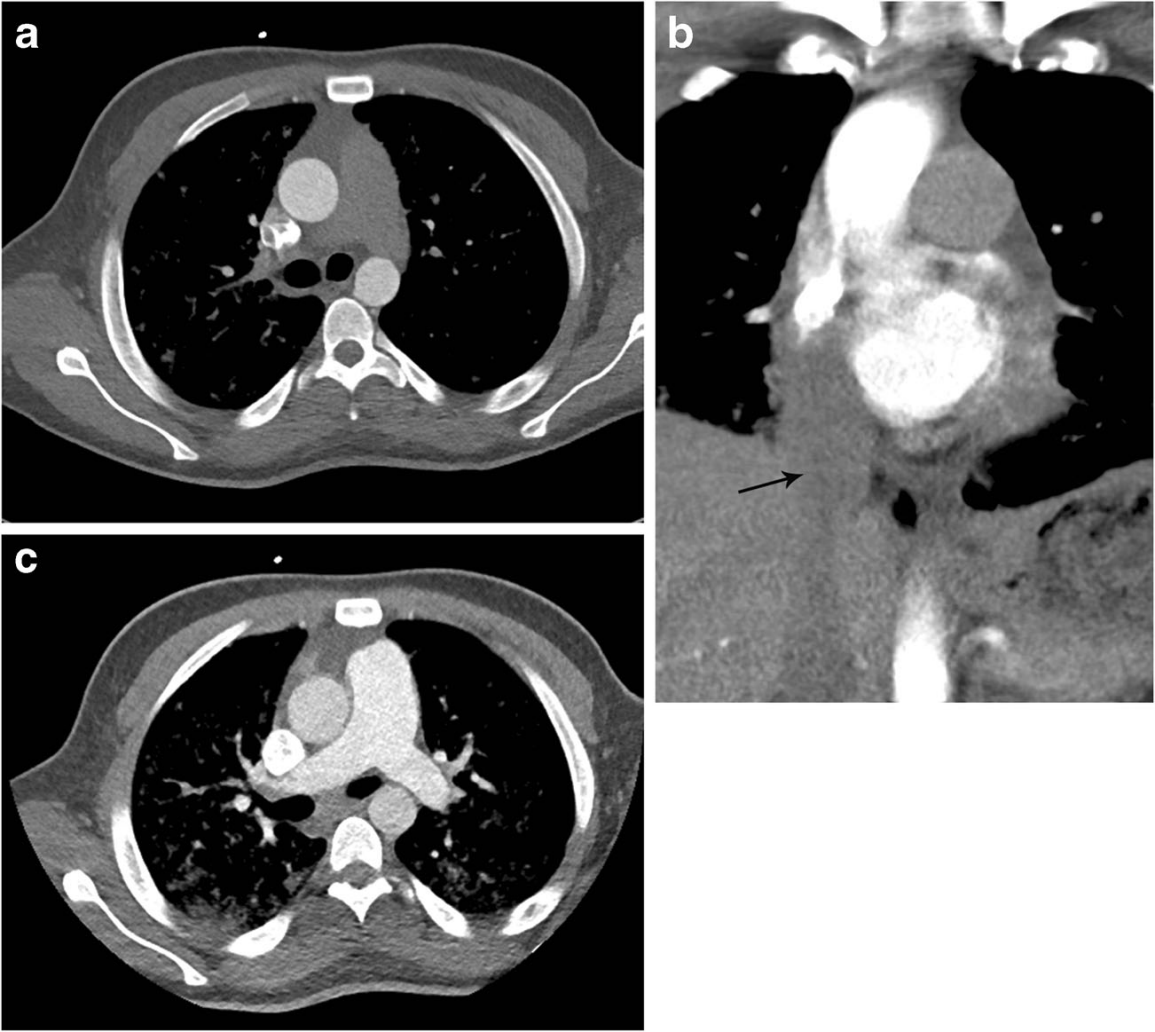
Transient interruption of contrast bolus results in suboptimal opacification of the pulmonary artery on initial contrast bolus, with subsequent diagnostic scan for pulmonary embolus after repeat injection using high pitch FLASH CTA. The initial CECT (**a**) shows poor opacification of the pulmonary artery despite high density contrast material within the aorta and SVC because deep inspiration resulted in increased venous return from the IVC and dilution of the contrast bolus in the right heart (**b**) at the time of scanning. Repeat injection using high pitch FLASH technique with free breathing (**c**) shows excellent opacification of the pulmonary artery

Considered a physiologic artifact, transient attenuation or interruption of the contrast bolus refers to disruption of the normal opacified contrast column secondary to return of unopacified venous blood via the inferior vena cava (IVC) in the setting of deep inspiration (Fig. 7a and b). There are two significant imaging consequences of this artifact: missing a true pulmonary embolus due to decreased opacification of the pulmonary artery or misinterpreting the decreased vessel attenuation as an embolus when it is not present. The pathophysiologic mechanism of this artifact is secondary to the normal variable inflow of blood to the right heart during inspiration. Referred to as the “abdominal-thoracic pump”, initial deep inspiration decreases intrathoracic pressure and increases intraabdominal pressure, acutely increasing venous return, favoring flow from the IVC over the superior vena cava (SVC), resulting in a bolus of nonopacified blood entering the right heart from the abdomen [19, 20].

This artifact should be considered when there is decreased opacification of multiple bilateral pulmonary arteries at the same level without vessel lumen distention: true pulmonary emboli typically present at various levels and normally expand the vessel lumen acutely [19]. In patients unable to hold breath, alternatively a free breathing high pitch flash CTA may be obtained [21] (Fig. 7c).

Imaging pearl: Techniques to overcome this artifact often rely on patient respiratory coaching, as the command “take a breath in and hold it” can lead some patients to take a rapid deep inspiratory breath, increasing the risk of transient attenuation of the contrast bolus. Alternate breathing instructions include requesting the patient to stop breathing or to take a slow gentle breath [20, 22].

### Cardiac causes of poor non-target vessel opacification

Sequential contrast opacification of central veins and cardiac chambers can be observed when bolus timing technique is used to identify contrast arrival. Normal sequence of enhancement follows right atrium, right ventricle, pulmonary artery, pulmonary vein, left atrium, left ventricle, and aorta. Earlier opacification of a distal chamber may be an indicator of intra or extra-cardiac shunt. When using bolus tracker technique, failure to adequately opacify the target vessels to reach the threshold needed for triggering the scan may also be an indication of decreased cardiac pump function.

**Fig. 8:**
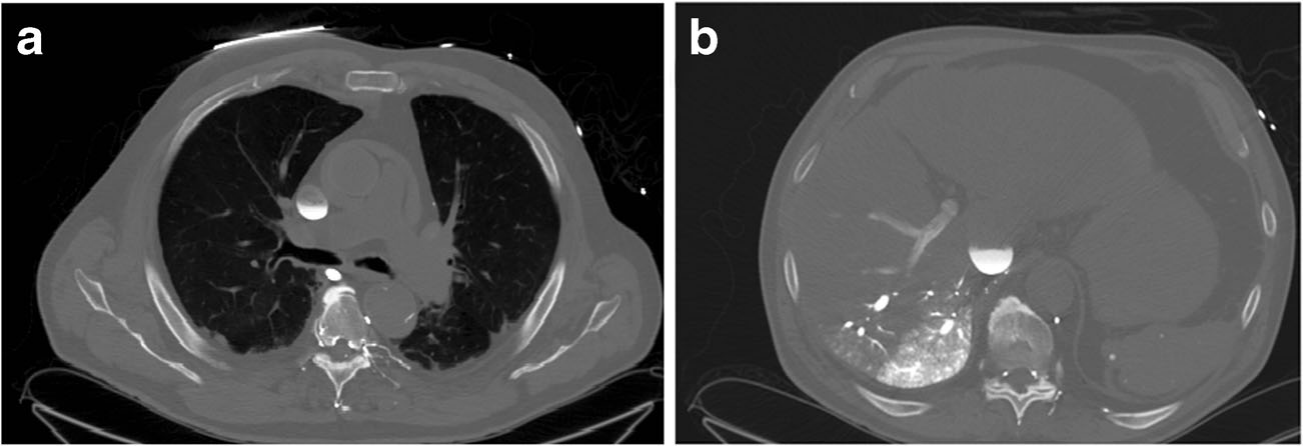
Asystole with no cardiac output. Patient with cardiac arrest at the time of CTA for acute aortic syndrome. Axial CTA at the level of the carina (**a**) shows no opacification of pulmonary artery, ventricles, or aorta. The CT technician subsequently called the radiologist to check the images and ask for a repeat injection after identifying abnormal contrast enhancement. Review of the axial images in the upper abdomen (**b**) reveal contrast reflux into IVC, dependent hepatic veins, and a blood-contrast level in the IVC. Contrast opacifies the right portal vein secondary to backflow from hepatic vein into portal vein. Contrast opacification is of the dependent vasculature only. This prompted initiation of cardiopulmonary resuscitation and calling the code team. See also Movie 1

Although the real incidence of cardiac arrest at the time of CT is not known, it is probably not rare [23]. In these patients, the contrast is distributed almost entirely in the venous system with no opacification of the right ventricle, pulmonary artery or aorta and indicates circulatory dysfunction (Fig. 8a and b). There may be retrograde opacification of IVC, hepatic veins, and even portal vein with dependent pooling of the contrast forming a blood-contrast level (Movie 1) [24]. The distribution of contrast medium is now being determined by the push from the power injector and the viscosity of the contrast medium. These patients are likely hemodynamically unstable at the time of presentation and may be on cardiopulmonary monitoring which should be evaluated by the attending radiologist. If the patient is not being monitored, and when such a finding is seen on a nondiagnostic CTA, it is imperative to call the code team and immediately begin cardiopulmonary resuscitation rather than planning for a reinjection.

**Fig. 9:**
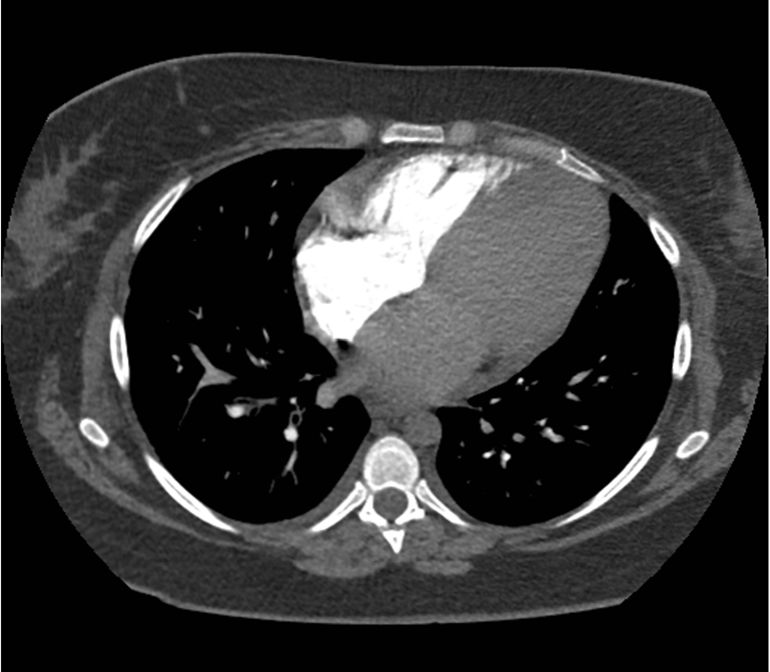
Cardiomyopathy resulting in poor contrast opacification of the left atrium, left ventricle and aorta on a thoracic CTA being obtained to evaluate for pulmonary embolism on a 64 slice CT. CECT demonstrates excellent opacification of the right heart, but poor opacification in the left heart related to prolonged pulmonary circulation time in a patient with left ventricular systolic dysfunction. BNP was immediately obtained and was elevated at 23,000, echocardiogram obtained within the next 4 h demonstrated a left ventricle EF of 22 %

**Fig. 10:**
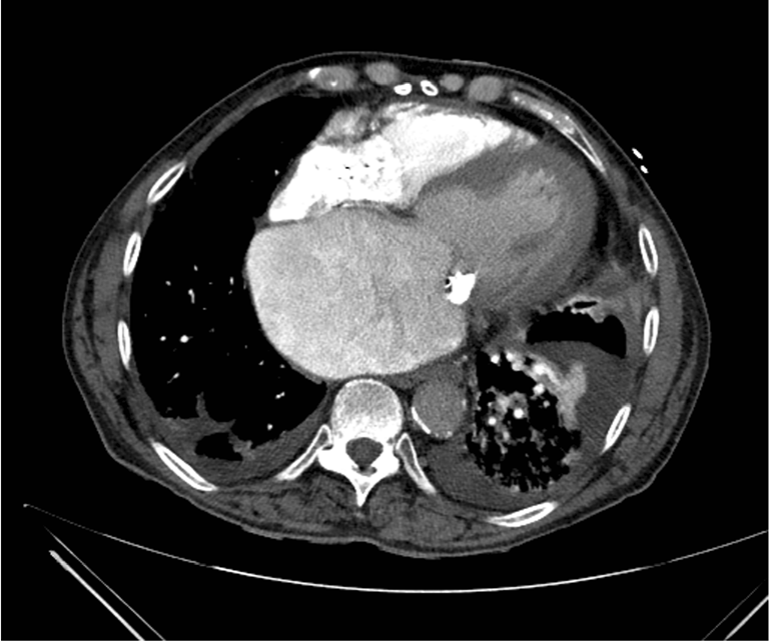
Mixing artifact in the left atrium in a patient with severe mitral regurgitation. CECT shows marked left atrium enlargement (*arrow*) with mixing of opacified blood coming from pulmonary veins and non-opacificed blood from the left ventricle due to severe mitral regurgitation

A less dramatic, but equally important observation may be seen in patients with congestive heart failure with resultant poor or no opacification of left cardiac chambers and aorta during a CT pulmonary angiogram (Fig. 9). The likely explanation for these findings can be increased pulmonary transit time. When using a scanner with shorter acquisition time, non target vessel enhancement may be less than expected, and these vessels should be interpreted with caution. Contrast-blood mixing artifacts are often seen in the right atrium, right ventricle and pulmonary artery during a pulmonary artery CTA due to unopacified blood returning from the IVC. These are, however, not commonly seen in left atrium or left ventricle, and whenever seen should be considered abnormal (Fig. 10).

**Fig. 11:**
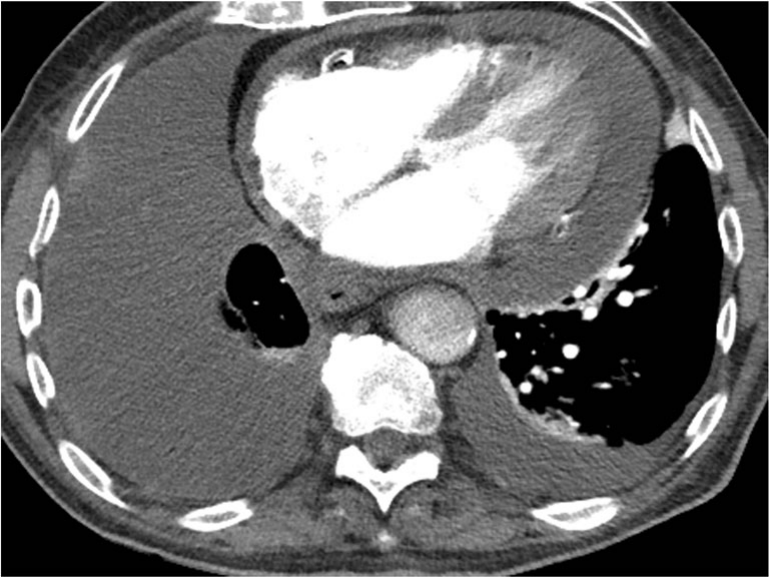
Pericardial tamponade on CECT. Axial CTA shows moderate pericardial effusion and heterogeneous enhancement of descending aorta with blood contrast layering. This is indicative of poor stroke volume from tamponade

Decreased systolic function of left ventricle can result in dependent contrast pooling and layering in the aorta [25]. This is likely due to decreased stroke volume with resultant contrast blood pooling with dependent layering of the higher viscosity contrast. Such dependent contrast pooling in descending aorta can also be seen in patients with acute cardiac tamponade, likely due to decreased stroke volume (Fig. 11).

Imaging Pearl: In patients with known heart failure, test bolus can be more useful in identifying time to peak enhancement, which can be delayed compared to contrast arrival time. Use of delayed images after 30 s can help differentiate soft plaque/thrombus from slow flow when dependent pooling is seen.

### Differential enhancement of pulmonary arteries

**Fig. 12:**
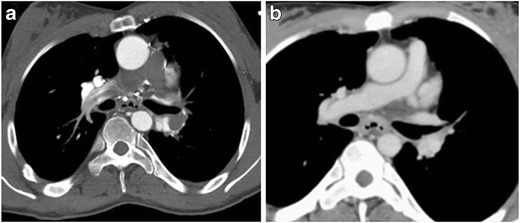
Two images from a CTPE study obtained in a patient with tricuspid atresia and bidirectional cavopulmonary connection demonstrate poor opacification of right pulmonary artery and non-enhancement of left pulmonary artery (**a**). Findings could represent thrombus. Delayed images (90 s) demonstrate complete opacification of both pulmonary arteries and the intracardiac Fontan (**b**, also see Movie 2). In addition, notice the higher attenuation in the right superior and inferior pulmonary veins compared to the adjacent pulmonary artery. This is similar to contrast attenuation in SVC. Careful attention demonstrates mediastinal venous collaterals draining directly into the pulmonary veins forming an extra-cardiac right to left shunt

**Fig. 13:**
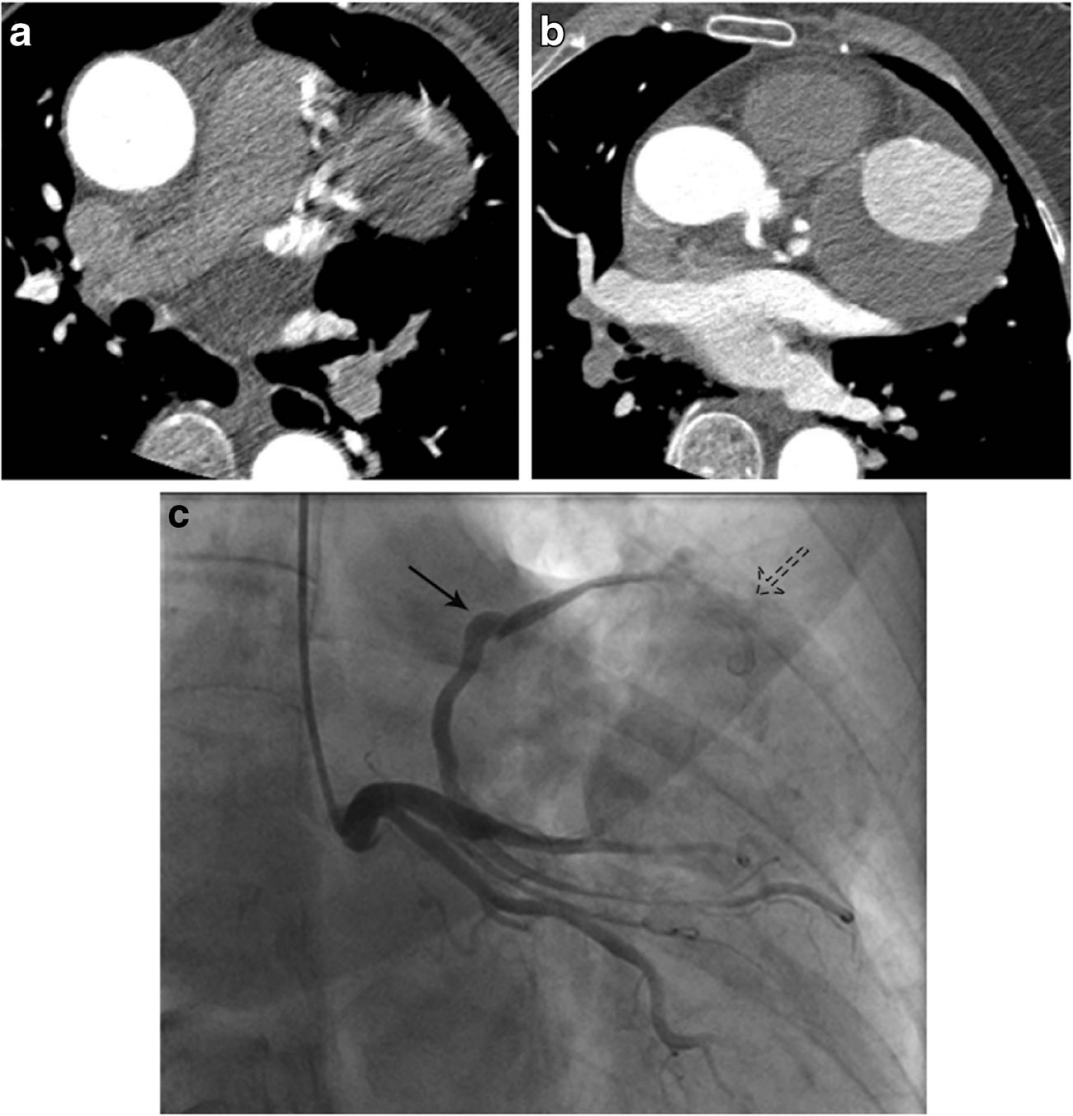
Axial CT images from a patient with coronary artery to pulmonary artery fistula, which results in partial opacification of the main pulmonary artery while contrast is maximally opacifying the aorta, seen on axial CTA (**a**, **b**) and conventional angiography (**c**). On conventional angiography, the fistula is seen opacifying on the arterial phase (*arrow*). The result is a systemic to pulmonary artery shunt. Notice the large thrombus in the A-V malformation abutting the main pulmonary artery

**Fig. 14:**
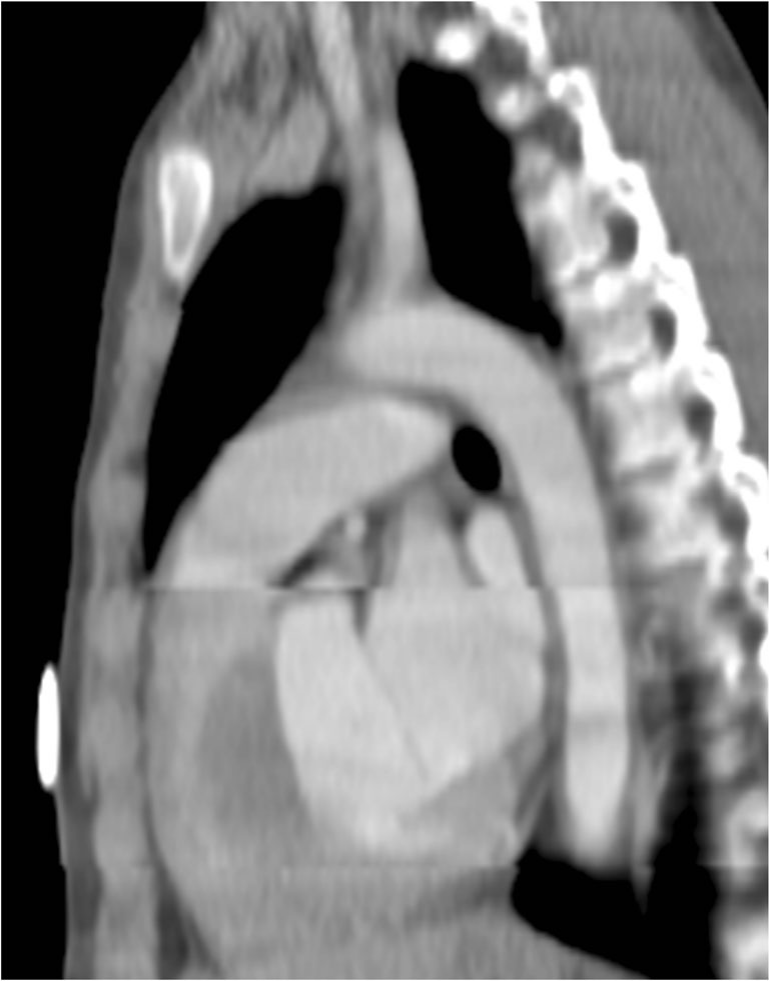
Coronal reformat from a thoracic CTA in a 13-year-old patient with mid aortic syndrome demonstrates step ladder artifact in the pulmonary artery as well as descending aorta. Note the differential enhancement in the right ventricle outflow tract vs. main pulmonary trunk. Also note the differential enhancement in descending aorta

Differential enhancement of pulmonary arteries during a pulmonary artery CTA can be seen in patients with Fontan circulation (Fig. 12), extra-cardiac shunts such as patent ductus arteriosus, bronchial artery, or coronary artery fistulas (Fig. 13), and when using prospective ECG triggered CTA (Fig. 14). Cavopulmonary shunts that connect the caval and pulmonary circulation are performed in patients with single ventricle physiology. Glenn shunt is performed as the second stage of surgical repair and involves anastomosis between the SVC and the right pulmonary artery, which can either be unidirectional or bidirectional. Fontan shunt is performed as the third stage of ventricular repair and involves anastomosis between the IVC and the left pulmonary artery. In the lateral tunnel Fontan, the right atrial wall is used to create a baffle, whereas in an extra-cardiac Fontan, a conduit is used to connect IVC blood to the pulmonary artery. In classic Fontan, the right atrium and the pulmonary artery are anastomosed. Total cavopulmonary connection involves a Glenn shunt connecting SVC to the right PA and Fontan shunt connecting IVC to left PA. CT angiography in these patients to visualize the pulmonary arteries or the conduits themselves is challenging since the SVC flow is directed to the right lung and the IVC flow is directed to the lung (Fig. 13a). Hence, injecting contrast only through the arm will not result in opacification of the left pulmonary arteries and injection through the lower extremity will not result in opacification of right pulmonary arteries, resulting in non-diagnostic studies [26]. This is important since there is a higher risk of pulmonary thromboembolism (3–19 %) in these patients [27]. Furthermore, due to the absence of pumping action of right ventricle, there is passive laminar flow of Fontan circulation, which causes inhomogeneous enhancement, particularly within the conduit [28]. It has been shown that 13 % of these patients have mural thrombus in the extracardiac conduit [27], even without symptoms, which may be missed with suboptimal studies [27] Solutions for this are (1) Simultaneous upper and lower extremity (femoral vein/foot vein) injections at 4–5 mL/s, so that both the SVC and IVC are opacified simultaneously [27]. However, the arrival of contrast media may not always be simultaneous due to different resistance, collaterals, and flow velocities. (2) Two-phase CT angiography, with both arterial and delayed venous phases (Fig. 12b, Movie 2) [26]. Delayed phase scan only. Delayed phase scan at 3 min has been shown to be good in visualizing entire vasculature during recirculation, regardless of the intravenous route or surgical technique [28]. Some authors use a 1-min delay provided the injection is antecubital due to shorter distance to pulmonary artery and in patients with cavopulmonary connections than atriopulmonary connections [28].

### Differential enhancement of thoracic aorta

Differential enhancement of ascending and descending aorta during a thoracic aortic CTA can be seen by using a prospectively triggered acquisition, coarctation, large aneurysms, and dissections. When prospective ECG gating is used (Fig. 14), there may be a delay between consecutive axial acquisitions which is exaggerated in the presence of irregular heart rate. This can lead to differential enhancement in different segments of the aorta, which merely indicates different contrast density at different time points. There is no solution to this artifact once acquired, but this can be avoided by using spiral instead of axial acquisitions.

**Fig. 15:**
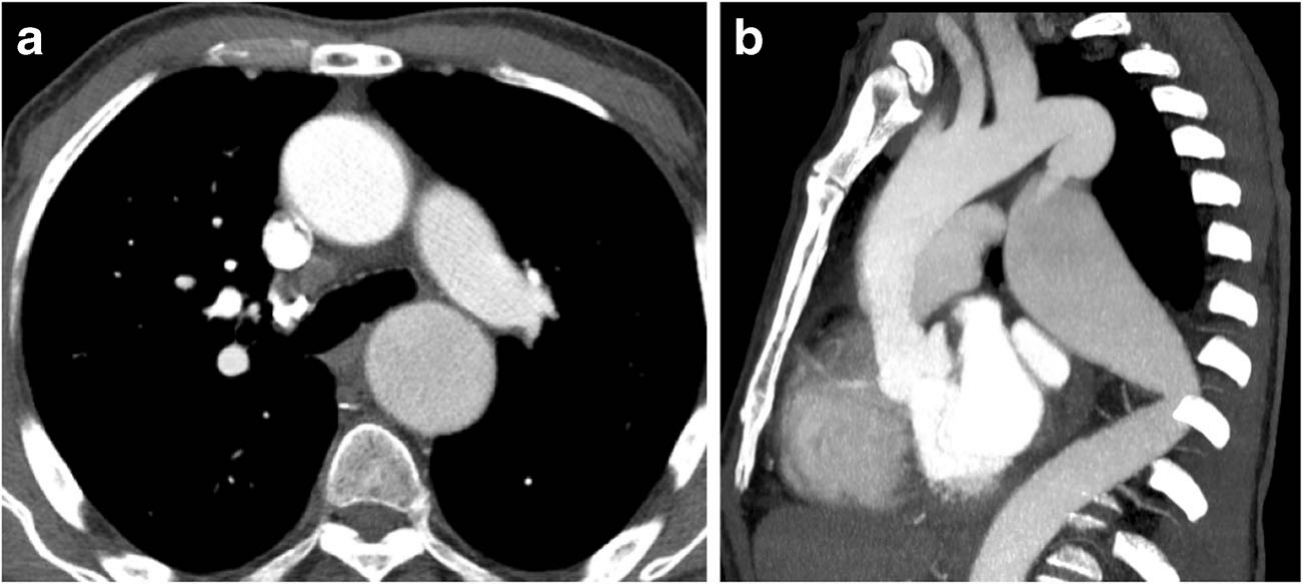
Axial CT image in 46-year-old patient with 20-year H/O essential hypertension presenting with chest pain being evaluated for aortic dissection demonstrates differential enhancement of the ascending and descending aorta due to severe stenosis from coarctation. Sagittal CECT MIP image demonstrates differential opacification of the aorta proximal and distal to the aortic coarctation with post-stenotic dilatation. Pressure gradient measured during catheter angiography was 20 mm Hg across the stenosis

Differential aortic enhancement can also be seen in patients with coarctation of aorta (Fig. 15). This is due to dilution of contrast within the blood pool of the post stenotic dilated aortic lumen. Unless sagittal images are also reviewed, this subtle sign may be the only significant clue seen on axial CTA images.

**Fig. 16:**
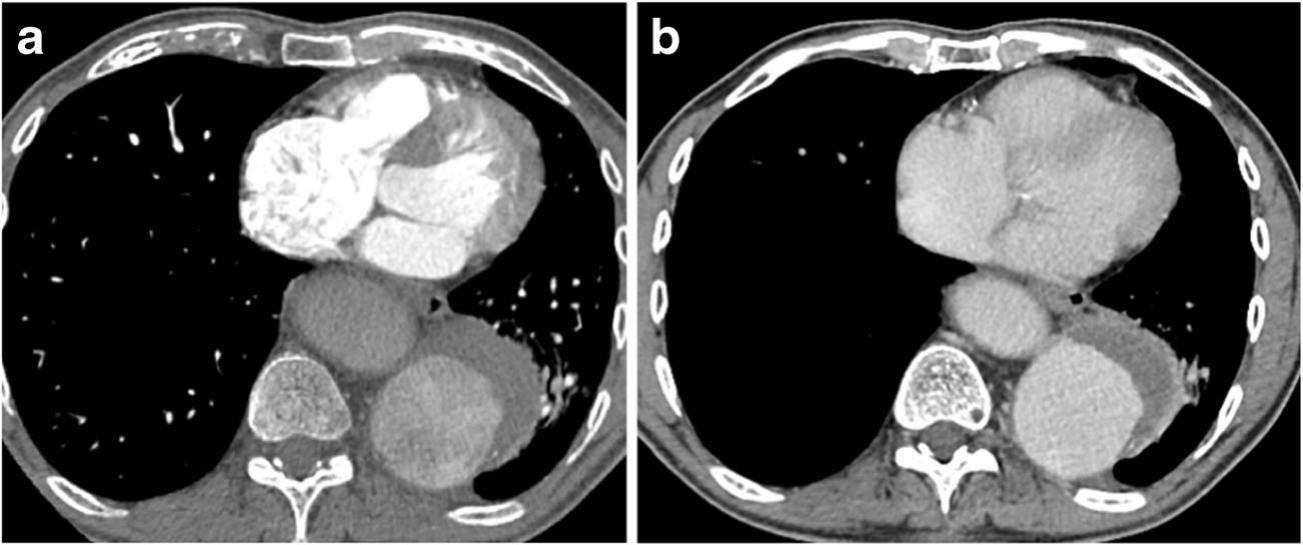
Mixing artifacts in the true lumen of the descending aortic aneurysm with Type B aortic dissection which resolve on delayed phase acquisition. Axial CECT (**a**) shows a type B dissection with mixing of contrast in the true lumen (*arrow*). Delayed phase CECT in the same patient (**b**) shows resolution of the mixing artifact. Note the complete nonopacification of false lumen. This phase allows more accurate estimation of the extent of the false lumen thrombus and slow flow

Mixing artifacts can be seen in large aortic aneurysms and should not be confused with a thrombus (Fig. 16a). Delayed images can help in opacification of the lumen (Fig. 16b).

Differential enhancement of false lumen of an aortic dissection can also be due to delayed opacification due to higher inherent luminal pressures. This should not be confused with a thrombus. In type B dissection, identification of false lumen thrombus can be overestimated by first pass CTA/MRA. True estimation of this false lumen thrombosis after aortic dissection is important as this can be important for prognosis [29]. Delayed phase acquisition is recommended for a more accurate estimation of true extent of false lumen thrombus vs. slow flow. Aortic dissection with partial thrombosis of the false lumen has a significantly higher annual aortic growth rate when compared with those patients with complete thrombosis of the false lumen [30].

Imaging pearl: In patients with known aortic aneurysm, ROI for test bolus or bolus tracking should be placed in that portion of the aorta which has the largest diameter.

### ECMO

**Fig. 17:**
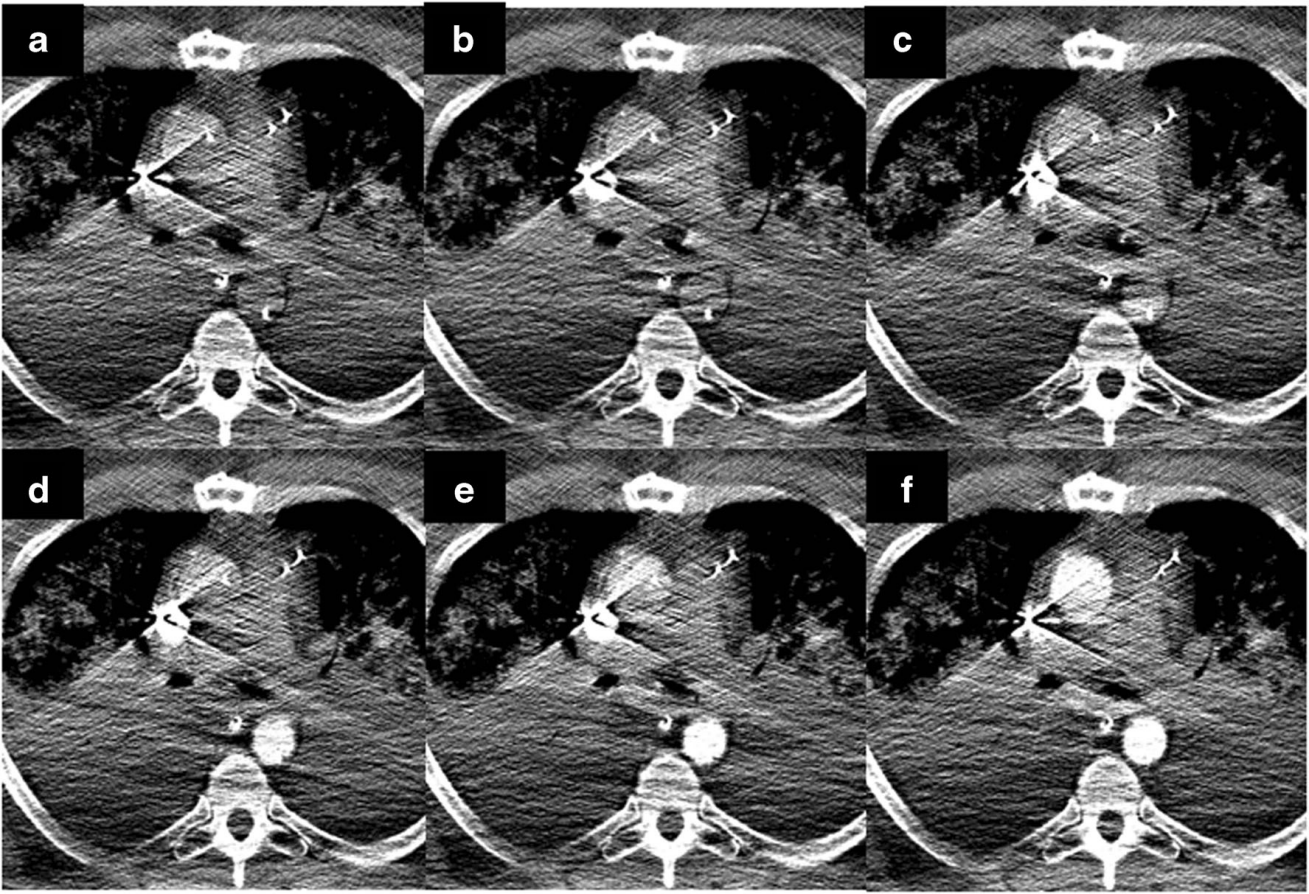
Sequential axial CT images (**a**–**f**) from a test bolus obtained at the level of main pulmonary artery. Notice the altered pattern of contrast flow. In a normal patient, contrast injected from the upper extremity first opacifies the SVC, followed by the right atrium, right ventricle, pulmonary artery, pulmonary vein, left atrium, left ventricle, ascending aorta, and finally the descending thoracic aorta. In this case we see an altered pattern of contrast flow: first SVC, followed by minimal opacification of the pulmonary artery and ascending aorta, dense opacification of the descending aorta followed by dense opacification of the ascending aorta. There is insufficient opacification of the pulmonary circulation due to siphoning of contrast by ECMO

Extracorporeal membrane oxygenation or ECMO is increasingly being used in adults for pulmonary or cardiopulmonary support in not just pediatric, but also adult patients with severe respiratory failure or following failure to wean from cardiopulmonary bypass after cardiac surgery [31]. The hemodynamics of flow in these patients, especially those on a venoarterial ECMO, are altered, with retrograde flow occurring in the access artery and in case of femoral artery access, in the aorta [32]. This can lead to variable enhancement pattern (Fig. 17a–f) of aorta, poor opacification of cardiac chambers, and suboptimal enhancement of the pulmonary vessels.

**Fig. 18:**
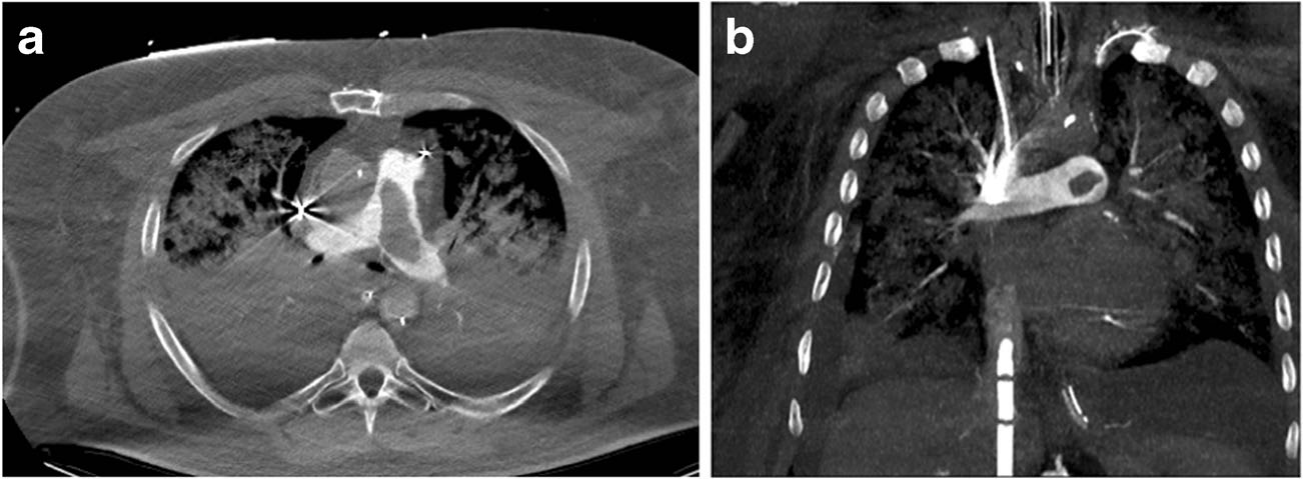
Axial CTA images from the same patient as in Fig. 17 obtained after the ECMO circuit was put on minimal flow status for a short period (25 s) to allow for near physiologic circulation. Axial (**a**) and coronal reformatted pulmonary CTA (**b**) subsequently demonstrates a large central pulmonary embolism

Imaging Pearl: Different approaches have been suggested to perform contrast-enhanced CTA in patients on ECMO: injection into the arterial cannula of the ECMO after the membrane oxygenator or into the venous line distal to the membrane oxygenator [33]. In our experience, slowing the flow of the circuit to the minimal flow rate that would prevent thrombus formation for the duration of the scan (15–20 s) has worked well in cases of suspected pulmonary embolism (Fig. 18).

### Central venous catheters

Central venous catheters are often used for contrast injection. Although there are safety issues related to this such as the risk of catheter rupture, fragmentation, or thromboembolism, these devices can be safely used if appropriate precautions including manufacturer specifications are followed [34]. Studies have shown that vascular enhancement is superior with central venous catheter injections compared to peripheral route injections due to the short time to peak enhancement facilitated by shorter travel distance for contrast bolus. There is also reduced individual patient variability [8]. However, the contrast injection is typically performed slower than peripheral routes due to safety concerns. Since the contrast media will directly opacify the lower SVC or the right atrium and the subsequent cardiovascular structures, the upper SVC and other veins will not be adequately opacified in the first pass as with a peripheral route injection. Hence, if venous visualization is the primary clinical objective, a delayed phase should be obtained in addition to the arterial phase.

Imaging Pearl: Manufacturer recommendations for the central venous catheter that is being used should be adhered to for peak flow rate. A delayed phase, 40 s acquisition can help in identifying any thrombus, vegetation, or fibrin sheath attached to the catheter.

## Conclusion

Non-diagnostic thoracic CTAs are frequently encountered in clinical practice. Careful interpretation of power injector graphs, location of region of interest to trigger the scan, and pattern of contrast flow can help determine the cause. This can help in planning a reinjection and obtain a diagnostic scan. Pattern of opacification of non target vessels can be useful in understanding the hemodynamic status of the patient and correctly identifying life threatening conditions such as tamponade, heart failure, and asystole.

## Electronic supplementary material

Below are links to the electronic supplementary material.

Movie 1 Axial CTA images being obtained in a patient suspected for acute aortic syndrome and found to be in asystole with no cardiac output. CTA shows contrast reflux into IVC and dependent hepatic veins and blood-contrast level in the IVC. Contrast also opacifies the right portal vein secondary to backflow from hepatic vein into the portal vein. ROI over aorta never reaches threshold to trigger the scan. (MOV 563 kb)

Movie 2 Delayed phase axial CT from a CTPE study obtained in a patient with tricuspid atresia and bidirectional cavopulmonary connection demonstrate complete opacification of both pulmonary arteries and the intracardiac Fontan. (AVI 5728 kb)
